# ATLAS-SCALE SINGLE-CELL DATA SCIENCE REVEALS SIGNATURES OF AGING AND REJUVENATION

**DOI:** 10.1093/geroni/igad104.1427

**Published:** 2023-12-21

**Authors:** Robert Palovics

**Affiliations:** Stanford University, Stanford, California, United States

## Abstract

The use of recent high-throughput transcriptomics technologies allows us for the first time to study aging at an unprecedented granularity and temporal resolution. This talk aims to provide insights into aging and heterochronic parabiosis, a model for systemic rejuvenation, by studying an atlas-scale single-cell dataset encompassing >100k cells across 20 organs and 50 cell types in young and aged mice as well as in parabionts. We will explore the age mimicking and age reversing effects of parabiosis within each cell type, identify parabiosis mediated rejuvenation signatures that appear throughout the whole body, and present a structural overview of aging and parabiosis across different organs.

